# Dynamics of growth factor production in monolayers of cancer cells and evolution of resistance to anticancer therapies

**DOI:** 10.1111/eva.12092

**Published:** 2013-08-15

**Authors:** Marco Archetti

**Affiliations:** School of Biological Sciences, University of East AngliaNorwich, UK

**Keywords:** anticancer therapy, cancer, cooperation, evolutionary game theory, growth factors, heterogeneity, polymorphism, public goods, somatic evolution

## Abstract

Tumor heterogeneity is well documented for many characters, including the production of growth factors, which improve tumor proliferation and promote resistance against apoptosis and against immune reaction. What maintains heterogeneity remains an open question that has implications for diagnosis and treatment. While it has been suggested that therapies targeting growth factors are robust against evolved resistance, current therapies against growth factors, like antiangiogenic drugs, are not effective in the long term, as resistant mutants can evolve and lead to relapse. We use evolutionary game theory to study the dynamics of the production of growth factors by monolayers of cancer cells and to understand the effect of therapies that target growth factors. The dynamics depend on the production cost of the growth factor, on its diffusion range and on the type of benefit it confers to the cells. Stable heterogeneity is a typical outcome of the dynamics, while a pure equilibrium of nonproducer cells is possible under certain conditions. Such pure equilibrium can be the goal of new anticancer therapies. We show that current therapies, instead, can be effective only if growth factors are almost completely eliminated and if the reduction is almost immediate.

## Introduction

### Tumor heterogeneity

Heterogeneity of cells within a tumor is well documented for many types of cancers and many distinguishable phenotypes (Marusyk and Polyak 2010) and has important implications for disease progression (Maley et al. 2006), diagnosis, and therapeutic responses (Dexter and Leith 1986). As diagnostic biopsies sample only a small region of the tumor, treatments based upon such samples might not be effective against all tumor cells. Understanding the origin, extent, and dynamics of tumor heterogeneity therefore is essential for the development of successful anticancer therapies.

A basic question about heterogeneity is still unsolved (Merlo et al. 2006): how can more than one clone stably coexist in a neoplasm? Given that the development of cancer is a process of clonal selection (Cairns 1975; Nowell 1976; Crespi and Summers 2005; Merlo et al. 2006; Greaves and Maley 2012) in which cells compete for resources, space, and nutrients, one would predict that a mutant clone with a fitness advantage should drive other clones extinct and go to fixation. Current explanations for the maintenance of heterogeneity include the possibility that different clones are evolutionarily neutral (Iwasa and Michor 2011), specialize on different niches (Nagy 2004; Gatenby and Gillies 2008) or are not in equilibrium (Gonzalez-Garcia et al. 2002), or that mutations have small effect (Durrett et al. 2011); which, if any, of these mechanisms are at work in neoplasms remains an open question (Merlo et al. 2006).

Here, we show that stable heterogeneity for the production of growth factors arises as a direct consequence of the fact that growth factors are nonlinear public goods. We develop a model of public goods production in the framework of evolutionary game theory and extend it to take into account specific features of the production of growth factors by cancer cells growing on a monolayer. We show how the evolutionary dynamics of the system can explain the maintenance of stable heterogeneity, how this affects the development of resistance to anticancer therapies that target growth factors, and its implications for the development of stable therapies.

### Game theory of cancer

Mathematical models of cancer were first developed to explain the relationship between the time of exposure to carcinogens and the number of tumors (Charles and Luce-Clausen 1942) to understand the number of mutations necessary to cause cancer (Nordling 1953) and the observed age-incidence patterns (Armitage and Doll 1954; Fisher 1958). The statistical study of age-incidence of hereditary versus sporadic cancers (Ashley 1969; Knudson 1971) was instrumental for the introduction of the idea of tumor suppressor genes. Following this line of research, most current models of cancer dynamics developed by ecologists and evolutionary biologists (Frank 2007; Byrne 1942) study the effect of mutations, selection, population size, and tissue architecture on the dynamics of cancer.

While game theory has often been mentioned (e.g., Gatenby and Maini 2003; Axelrod et al. 2006; Merlo et al. 2006; Basanta and Deutsch 2008; Lambert et al. 2011) as a promising avenue for cancer research, only a few studies actually develop game theoretical models of cancer. Tomlinson (1997) and Tomlinson and Bodmer (1997) used the game of chicken (Rapoport and Chammah 1966) [or hawk-dove game (Maynard Smith and Price 1973) or snowdrift game (Sugden 1986)] to describe interactions between cancer cells. The model has been extended to up to four types of cells, using different types of cancer as examples, including multiple myeloma, prostate cancer, glioma, and glioblastoma (Basanta et al. 2008a,b, 2011, 2012; Dingli et al. 2009; Gerstung et al. 2011). Interactions among cancer cells for the production of diffusible growth factors, however, are not pairwise, but multiplayer, collective interactions for the production of a public good (Archetti 2013a). It is known that results from the theory of two-player games cannot be extended to games with collective interactions, and that this can actually lead to fundamental misunderstandings (Archetti and Scheuring 2012).

### Growth factors as public goods

Consider a population of cells in which a fraction of the cells (producers: +/+) secrete a growth factor. If the benefit of this factor is not restricted to the producers, we can consider it a public good that can be exploited by all other individuals (or cells) within the diffusion range of the factor, including nonproducers (−/−). Public goods are studied in economics, where rational, self-interested behavior may lead to the overexploitation of common pool resources [the ‘tragedy of the commons’ (Hardin 1968)], and in evolutionary biology in cases like the production of diffusible molecules in microbes (Crespi 2001). Diffusible public goods raise a collective action problem: an individual can free ride on the goods produced by his neighbors. Why, then, do noncontributors not increase in frequency and go to fixation? What factors influence the production of these public goods?

Similar collective action problems arise during cancer development, where growth factors support tumor growth by protecting cells from apoptosis (for example, IGF-II), by stimulating the growth of new blood vessels (for example, VEGF), by impairing immune system reaction (for example, TGFβ), or by promoting the epithelial–mesenchimal transition. While cooperation for the production of growth factors has been shown directly only in one case (FGF) (Jouanneau et al. 1994), it stands to reason that many diffusible factors produced by cancer cells benefit producers and nonproducers (Axelrod et al. 2006). Self-sufficiency of growth factor production is one of the hallmarks of cancer (Hanahan and Weinberg 2000) and, like for other characters, there is evidence of heterogeneity in the ability to produce diffusible factors (Achilles et al. 2001; Marusyk and Polyak 2010). What maintains this heterogeneity? And what are the implications for anticancer therapies?

It has been suggested that treatments that attack growth factors may be less susceptible than traditional drugs to the evolution of resistance (Pepper 2012; Aktipis and Nesse 2013). Current drugs that target growth factors, however, like the anti-angiogenic drug Avastin, have a limited effect, with only a few months of overall survival extension (Amit et al. 2013). Limited theoretical analysis has been devoted to investigating the problem of the evolution of resistance to therapies (Aktipis et al. 2011). The rationale of this study is that analyzing the production of growth factors in cancer as a public goods game can explain both stable heterogeneity and the long-term failure of antigrowth factor therapies and reveal conditions that can lead to evolutionarily stable therapies.

### Public goods games

Archetti and Scheuring (2012) review public goods games (PGGs) in well-mixed populations and Perc et al. (2013) review PGGs in structured populations. The current literature on PGGs often assumes that the benefit of the public good is a linear function of the number of contributors (the N-person prisoner's dilemma: NPD). The simplest cases of nonlinear benefits, synergistic, and discounting benefits (Motro 1991; Foster 2004; Hauert et al. 2006), as well as threshold PGGs (in which a benefit is produced if a number of contributors is above a fixed threshold) (Archetti 2009a,b; Pacheco et al. 2009; Boza and Szamado 2010; see also Palfrey and Rosenthal 1984 for a similar model in economics) have been studied extensively (Archetti and Scheuring 2012). The benefit produced by growth factors, however, is likely to be a sigmoid function of the number of producer cells because the effect of enzyme production is generally a saturating function of its concentration (e.g., Hemker and Hemker 1969), specifically, a sigmoid function (Ricard and Noat 1986); signaling pathways often follow a highly nonlinear on–off behavior, which is a steep sigmoid function of signal concentration (e.g., Mendes 1997; Eungdamrong and Iyengar 2004). Similar nonlinearities are known in microbes (Chuang et al. 2010). Sigmoid PGGs are somewhat intermediate between linear and threshold PGGs, while synergistic/discounting PGGs can be thought of as special, degenerate cases of sigmoid PGGs (Archetti and Scheuring 2011). Linear, threshold, and synergistic/discounting benefits can lead to dramatically different dynamics and equilibria in multiplayer games (Archetti and Scheuring 2012); the dynamics and equilibria of multiplayer sigmoid PGGs in well-mixed populations have been described analytically only recently (Archetti 2013a).

While this literature analyses PGGs in well-mixed populations, the study of PGGs in spatially structured populations generally assumes linear benefits (the NPD). The few exceptions (see Perc et al. 2013) using nonlinear benefits in spatially structured populations assume, as is standard in the current approach, that each individual belongs to n different groups, each group centered on one of that individual's one-step neighbors, and that an individual's fitness is the sum of all the payoffs accumulated in all the groups she belongs to (Perc et al. 2013). While this assumption is reasonable for interactions in human social networks, it is not appropriate for modeling interactions in cell populations, where the growth factors produced by one individual can diffuse beyond one-step neighbors, and the benefit an individual gets as a result of the diffusible factors is a function of the number of producers within the diffusion range of the factor, not of all individuals belonging to her neighbors' group. The only exceptions to the use of the standard framework are Ifti et al. (2004) and Ohtsuki et al. (2007): they study the prisoner's dilemma (that is, a two-person game with a linear benefit function) on lattices in which the interacting group is decoupled from the update neighborhood. Here, we need to analyze the more general case of sigmoid benefits (rather than linear) with collective interactions (rather than pairwise). We will not assume a particular type of cancer but describe the dynamics of growth factors like insulin-like growth factor II (IGF-II) that confer a direct beneficial effect to the cells, for example by protecting against apoptosis. Other growth factors confer a benefit to the tumor indirectly by stimulating the development of blood vessels or the release of other growth factors by stromal cells. The dynamics of these growth factors would be more complex.

## Model

### The game

A cell can be a producer (+/+) or a nonproducer (−/−) of a growth factor. Producers pay a cost c that nonproducers do not pay (0 < c < 1). All cells (+/+ and −/−) benefit from the public good produced by all the cells in their group (of size n; this depends on the diffusion range of the factor – see below). The benefit for an individual is given by the logistic function V(j)=1/[1 + e^−s(j−k)/n^] of the number j of producers among the other individuals (apart from self) in the group, normalized as b(j)=[V(j)-V(0)]/[V(n)-V(0)]. The parameter k controls the position of the inflection point (k*→*n gives strictly increasing returns and k*→*0 strictly diminishing returns) and the parameter s controls the steepness of the function at the inflection point (s*→*∞ models a threshold public goods game; s*→*0 models an N-person prisoner's dilemma) (Archetti and Scheuring 2011). It is useful to define h=k/n.

### Evolution in spatially structured populations

We model a monolayer of cancer cells as a two-dimensional regular lattice obtained using a modification of the GridGraph implementation in Mathematica 8.0 (Wolfram Research Inc.) connecting opposing edges to form a toroidal network, to avoid edge effects. As in the standard approach, individuals occupy the nodes of the network (population size is fixed at 900) and social interactions proceed along the edges connecting the nodes. Differently from the standard approach, however, [in which an individual's group is limited to her one-step neighbors and an individual plays multiple games centered on each of her neighbors (Perc et al. 2013)], the interaction neighborhood and the update neighborhood are decoupled: a cell's group (of size n) is not limited to her one-step neighbors but is defined by the diffusion range (d) of the growth factor, that is, the number of edges between the focal cell and the most distant cell whose contribution affects the fitness of the focal cell. A cell's payoff is a function of the amount of factor produced by the group she belongs to. The process starts with a number of nonproducer cells placed on the graph; at each round, a cell x with a payoff P_x_ is selected (at random) for update (death) and a cell y (with a payoff P_y_) is then chosen among x's neighbors. Two types of update are used: in the deterministic case, if P_x _> P_y_, no update occurs, while if P_x _< P_y_, x will adopt y's strategy (unconditional imitation); in the stochastic case, replacement occurs with a probability given by (P_y_-P_x_)/M, where M ensures the proper normalization and is given by the maximum possible difference between the payoffs of x and y (Perc et al. 2013). Results are obtained averaging the final 200 of 1000 generations per cell, averaged over 10 different runs.

### Gradient of selection in well-mixed populations

In a finite population, the gradient of selection can be calculated following Traulsen et al. (2006). Sampling of individuals follows a hypergeometric distribution and the average fitness of +/+ and −/− can be written as, respectively


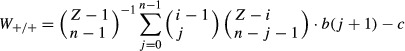



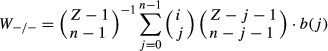


where i is the number of +/+ individuals in the population. Assuming a stochastic birth–death process combined with a pairwise comparison rule, two individuals from the population, A and B, are randomly selected for update. The strategy of A will replace that of B with a probability given by the Fermi function


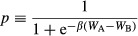


and the reverse will happen with probability 1-*p*. The quantity *β* specifies the intensity of selection (for *β *<< 1, selection is weak, and in the limit Z→∞ one recovers the replicator equation) (Traulsen et al. 2007). In finite populations, the quantity corresponding to the ‘gradient of selection’ in the replicator dynamics is given by


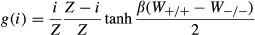


In an infinitely large population, the gradient of selection can be written as (Archetti and Scheuring 2012)


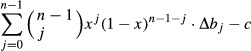


where Δ*b*_*j *_= *b*(*j* + 1)−*b*(*j*)

## Results

### Evolutionary dynamics of growth factor production

#### Decoupling the interaction and update networks

**Figure 1:**
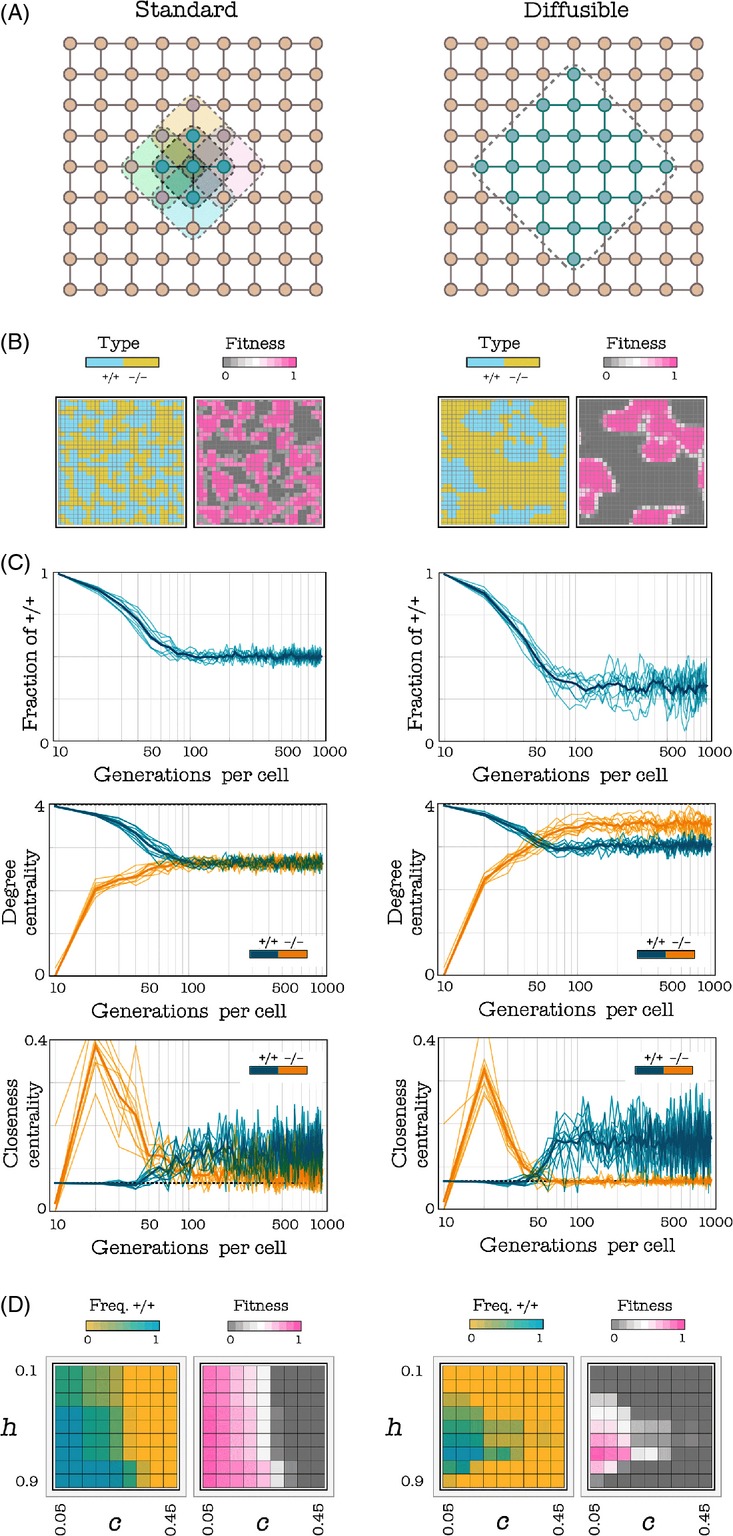
Growth factors as public goods. (A) In the standard approach, a cell's payoff is determined by the games played by the groups centered on that cell and on its one-step neighbors; in the case of diffusible factors, the group (the interaction neighborhood) is defined by the diffusion range (d) of the factor (here d = 3) and is larger than the update group (the one-step neighbors). (B) The structure of the population after 1000 generations per cell (c = 0.25, h = 0.5, d = 2, s = 20, deterministic update) (C) The change in frequency of +/+ cells, degree centrality, and closeness centrality of the +/+ and −/− subgraphs (c = 0.25, h = 0.5, d = 2, s = 20, deterministic update). (D) The equilibrium frequency of +/+ and average fitness as a function of h (the position of the threshold) and c (the cost of production) when 10 −/− cells are introduced in the population (d = 2, s = 20, deterministic update).

A comparison between the standard framework and the one used here (decoupling interaction and replacement networks) is possible if we assume that d = 2 (Fig. 1). Group size with d = 2 on a lattice with connectivity 4 is the same as the total number of individuals participating in the five PGGs in the standard approach, counting each individual only once (*n *= 25). In the standard approach, however, the focal individual contributes to all PGGs she is involved in, her one-step neighbors contribute to two PGGs that affect the focal individual, and her two-step neighbor contribute only to one PGG that affects the focal individual. In the case of diffusible factors instead, all individuals contribute equally to a single, larger PGG. Fig. 1 shows the differences between the two systems. Cooperation evolves for a wider parameter set in the standard approach than in the case of diffusible goods, and the fraction of producers is larger. This is not surprising, given the smaller group size implied by the standard approach. If d > 2 of course, the standard approach cannot be defined, and the two systems are not directly comparable. All the results are based on the new approach in which the interaction and replacement graphs are decoupled, and the diffusion range of the growth factor can be larger than 1.

#### Heterogeneity

**Figure 2:**
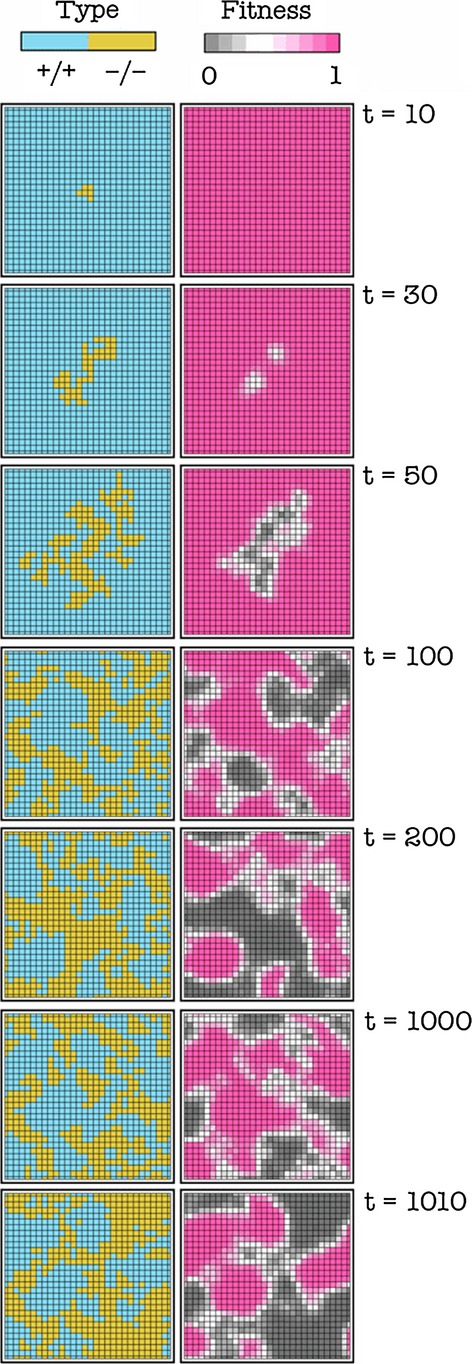
Dynamic heterogeneity. Snapshots of the population at different times (t is the number of generations per cell). The frequency of the two types remains relatively stable after about 100 generations per cell, but the position of +/+ and −/− cells changes. c = 0.02, h = 0.5, s = 20, d = 4; deterministic update.

When a nonproducer (−/−) is introduced in a population of producers (+/+), in most cases −/− cells increase in frequency and coexist with +/+ cells; this change in frequency of the two types is accompanied by a change in the relative position of the +/+ and −/− cells, as shown by the degree centrality (the number of neighbors) and the closeness centrality (the inverse of the sum of the distance to all other vertices) of the +/+ subgraphs (Fig. 1). In most cases after about 100 generations per cell, the frequencies remain relatively stable, even though the position of producers and nonproducers on the lattice continues to change (Fig. 2). In certain cases, the −/− type goes to fixation. The frequency of the two types, or the extinction of the +/+ type, depends on the diffusion range (d), the cost of growth factor production (c), the position of the inflection point of the benefit function (h), and the steepness of the benefit function (s), the update rule and the initial frequency of the two types, as described below.

#### Effect of the diffusion range

**Figure 3:**
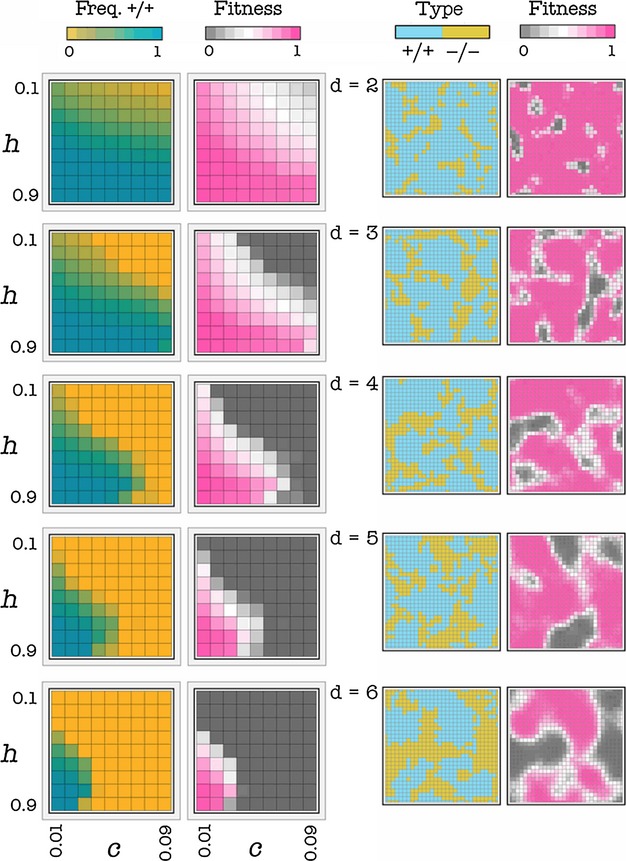
Effect of the diffusion range. Left: Each square in each plot shows the frequency of +/+ cells or the average fitness of the population (its growth rate) as a function of h (the position of the threshold) and c (the cost of production), for a given d (diffusion range) and for s = 20 (deterministic update). The frequency of +/+ cells and the average fitness are higher at intermediate levels of h and at low values of c. Both decline with increasing d, that is, increasing group size. Right: Snapshots of the population after 1000 generations per cell; c = 0.01, h = 0.5, s = 20, deterministic update.

Both the frequency of producers and fitness at equilibrium decline with increasing d (the diffusion range of the public good), that is, increasing group size (n); −/− cells form clusters whose size increases with d (Fig. 3). A short diffusion range enables a mixed equilibrium (coexistence of +/+ and −/− cells) for a larger set of parameters (higher c and more extreme h values). That is, a short diffusion range favors cooperation.

#### Effect of the initial frequencies

**Figure 4:**
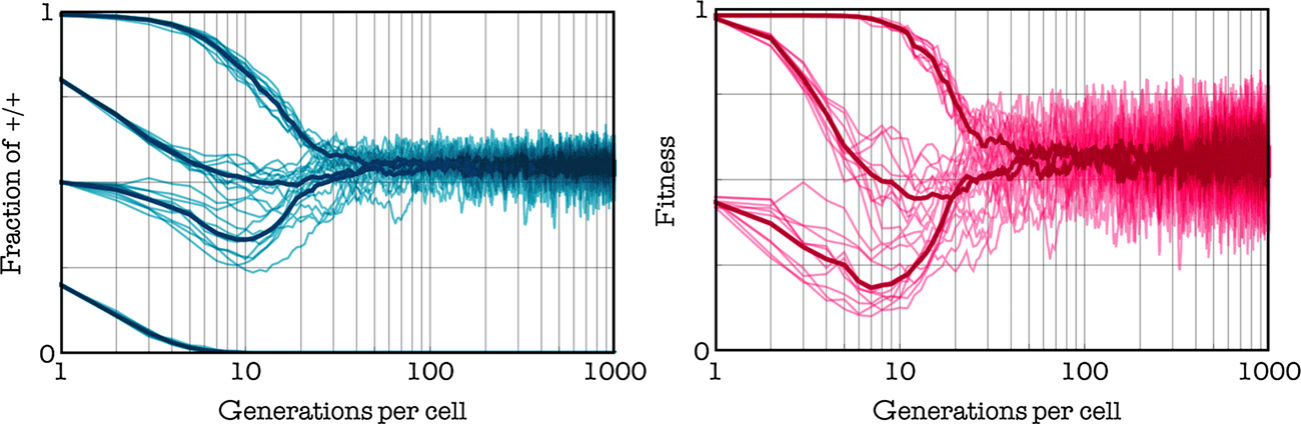
Stable heterogeneity. The change over time of the fraction of producers and of the average fitness of the tumor. At the stable mixed equilibrium, producers and nonproducers coexist, unless the initial fraction of producers is lower than an internal unstable equilibrium (here approximately 0.25). c = 0.02, h = 0.5, s = 20, d = 3.

**Figure 5:**
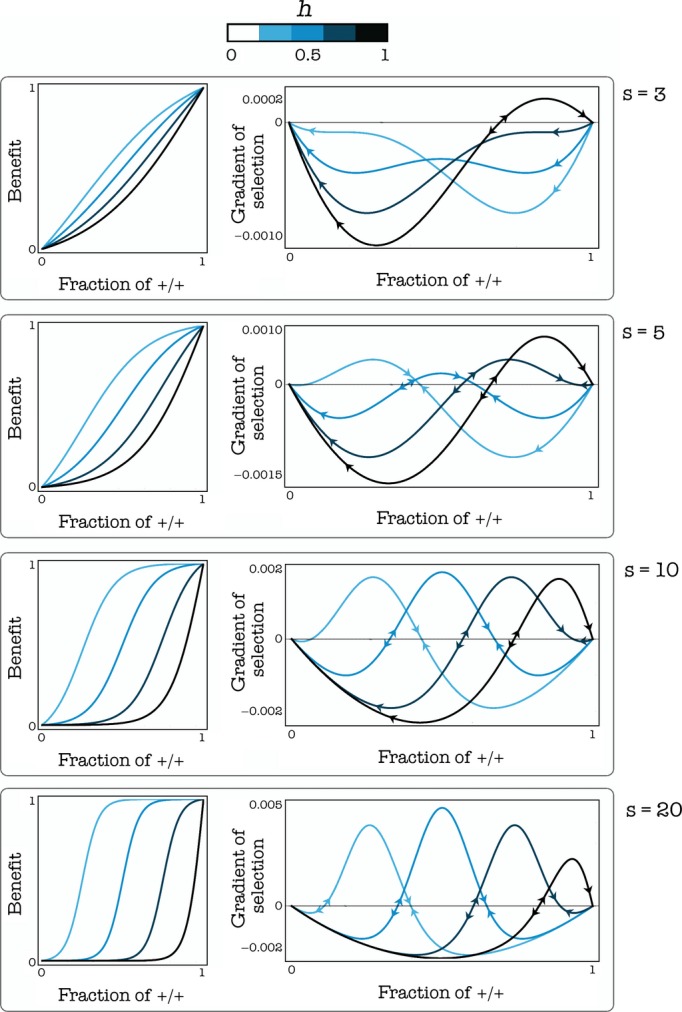
Evolutionary dynamics and equilibria. Left: The benefit functions and the gradients of selection for given values of s (the steepness of the benefit function) and h (the position of the threshold). The sign of the gradient of selection determines the dynamics (arrows show the direction of the change in frequency of +/+ individuals); equilibria occur where the gradient of selection is zero. c = 0.02; *β *= 1; d = 3

The stable equilibrium described above does not depend on the initial frequency of the two types (Fig. 4), unless the initial frequency of +/+ cells is below a certain threshold; in this case, the +/+ type goes extinct. In other words, the system has an internal stable equilibrium, to which the population evolves if and only if +/+ cells are above a critical threshold. Inspection of the gradient of selection shows the reason for the existence of mixed equilibria and bistability (Fig. 5): +/+ cells decrease in frequency when the gradient of selection is positive, that is when there are too few or too many +/+ cells; at intermediate frequencies of +/+ cells, however, +/+ cells have a selective advantage and can increase in frequency up to a stable mixture of +/+ and −/−. Below the unstable internal equilibrium, +/+ cells go extinct.

#### Effect of the benefit function

**Figure 6:**
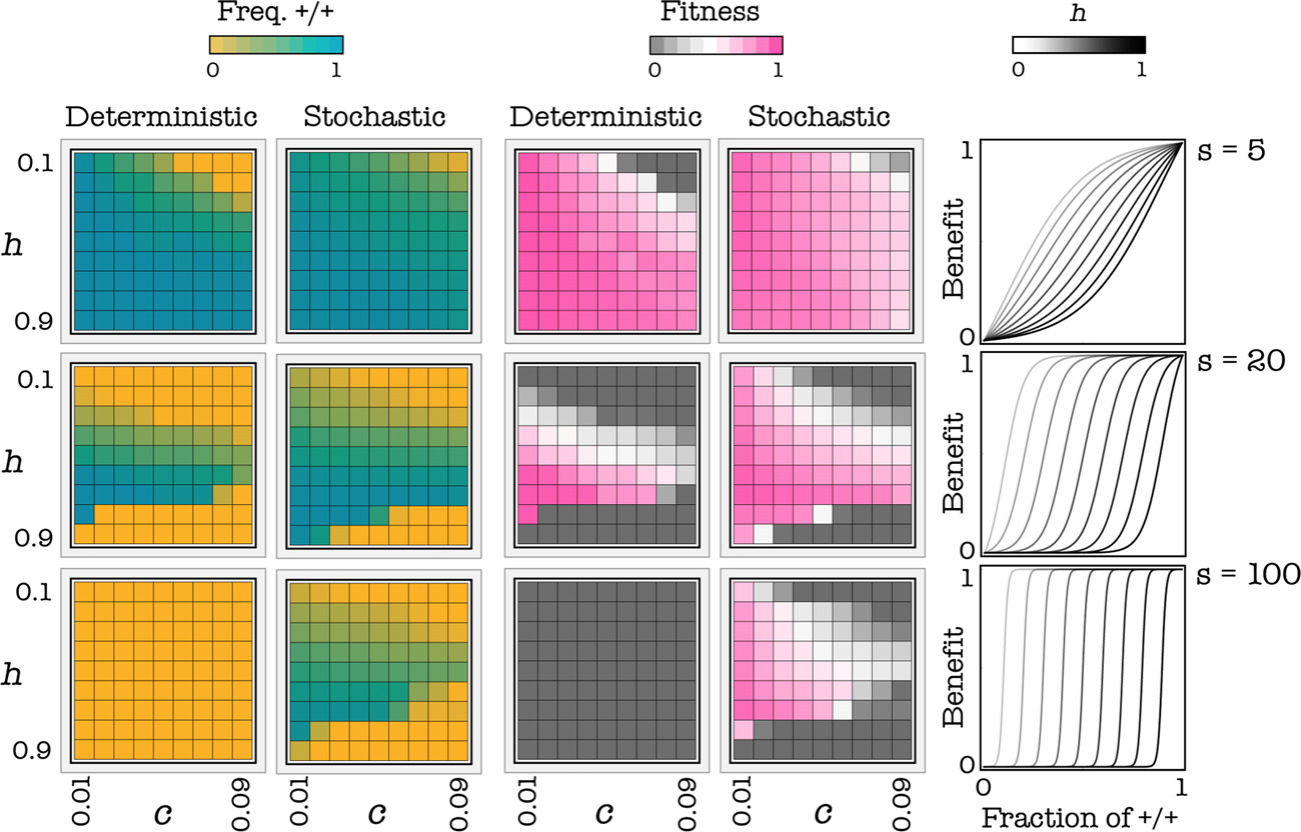
Effect of stochastic events. Each cell in each plot shows the frequency of +/+ cells or the average fitness of the population (its growth rate) as a function of h and c (the cost of production), for a given s at equilibrium. The deterministic update rule makes the internal stable equilibrium sensitive to stochastic fluctuations and therefore not robust when the benefit function is steep (high s); a more realistic stochastic update rule increases the robustness of the equilibrium. d = 3.

The internal stable equilibrium disappears for high values of h; the internal stable equilibrium disappears for low values of h, especially for low values of s. In the extreme case s*→*0, the game approaches the N-person prisoner's dilemma (the benefit function is a linear function of the frequency of cooperators), and both internal equilibria disappear. Both the frequency of producers and fitness are higher at intermediate levels of h (the position of the threshold) (Fig. 5). A shallow benefit function (low s) can favor cooperation, especially in the deterministic update rule. The reason can be understood more easily if we consider a step function with threshold k as an approximation of a very steep benefit function: in this case, it is convenient to be +/+ only when one is pivotal for the production of the public good, that is, only when there are exactly k-1 other +/+ cells. If the benefit function is a smooth sigmoid function, instead, it pays to be a +/+ even when not pivotal for reaching the threshold. In spatially structured populations, it easily happens that a mutant −/− arising in a group centered on one individual with few −/− also affect the number of +/+ in an adjacent group that was previously at equilibrium; in this other group, the frequency of +/+ will be now below the unstable equilibrium and therefore in the basin of attraction of the pure −/− equilibrium. This process is buffered in the stochastic update process but relatively fast in the deterministic update rule, which is therefore less permissive for the stability of cooperation. The deterministic update rule, therefore, is less conductive to cooperation than stochastic update, especially for very steep public good functions (Fig. 6).

#### Effect of the cost of production

As expected, increasing the cost of production (c) reduces both the frequency of producers and fitness at equilibrium (Fig. 5). A critical value of c exists above which no public goods production can be sustained and producers go extinct. This critical value is higher for intermediate values of the position of the threshold (that is, for h around 0.5) and for lower values of the diffusion range (d), and it also depends on the type of benefit function and update rule (Fig. 6)

### Evolutionary dynamics of resistance to therapies that target growth factors

#### Effect of therapies that increase the threshold

**Figure 7:**
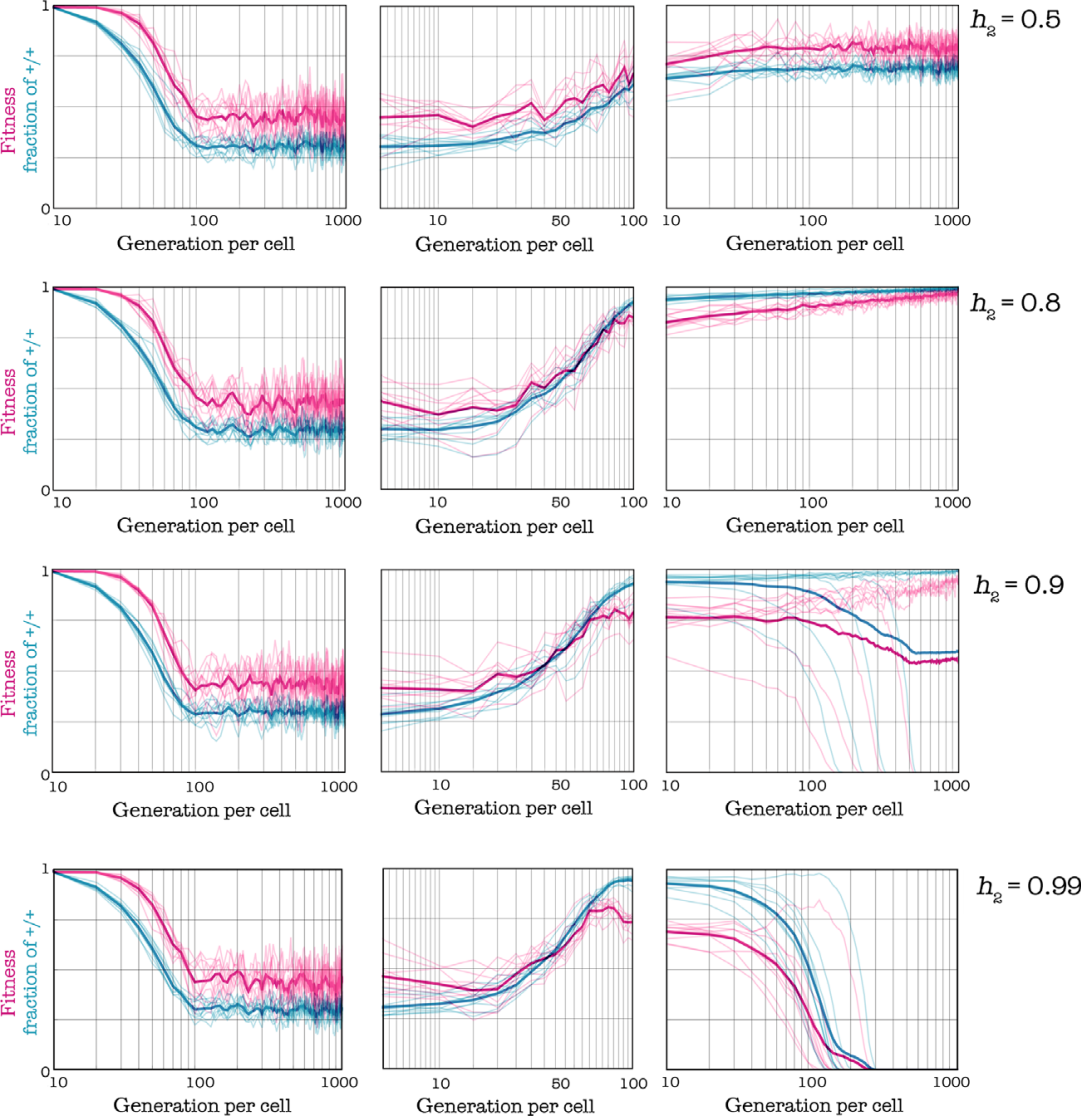
Effect of therapies that target growth factors. Reducing the amount of available growth factor increases the threshold from h_1_ = 0.3 to h_2_. d = 3, h_1_ = 0.3, c = 0.01, s = 20. In all cases, the shift from h_1_ to h_2_ occurs gradually, after 1000 generations per cell, in 100 generations.

An anticancer therapy that acts by impairing circulating growth factors will increase the amount of growth factors that the cells must produce to achieve a certain benefit, that is, it will increase the threshold h. Two results are possible. In the first case, the population adapts to the new threshold, that is, +/+ cells increase in frequency and fitness increases - the opposite of the scope of the drug; only if the threshold increase is substantial does the +/+ type go extinct (Fig. 7).

#### Effect of the speed of change

**Figure 8:**
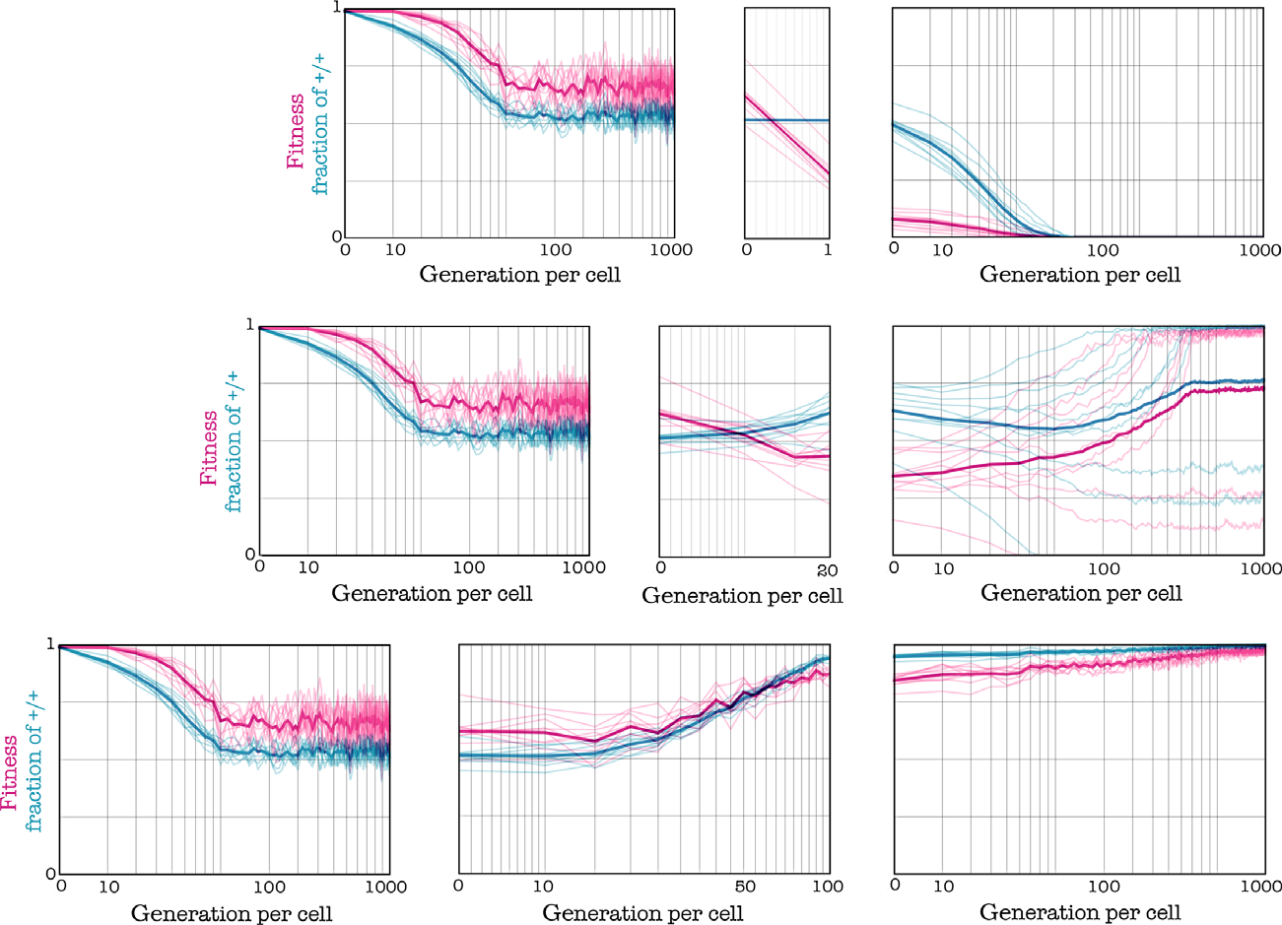
Importance of the speed of change. Reducing the amount of available growth factor increases the threshold from h_1_ = 0.4 to h_2_ = 0.8; d = 3, c = 0.01, s = 20. If the change occurs immediately (in the following generation), the +/+ type goes extinct; if the change takes 100 generation per cell to be completed, the population moves to a new equilibrium with a higher fraction of +/+ cell and higher fitness (the contrary of the desired effect); if the change takes 20 generations per cell, results are intermediate.

**Figure 9:**
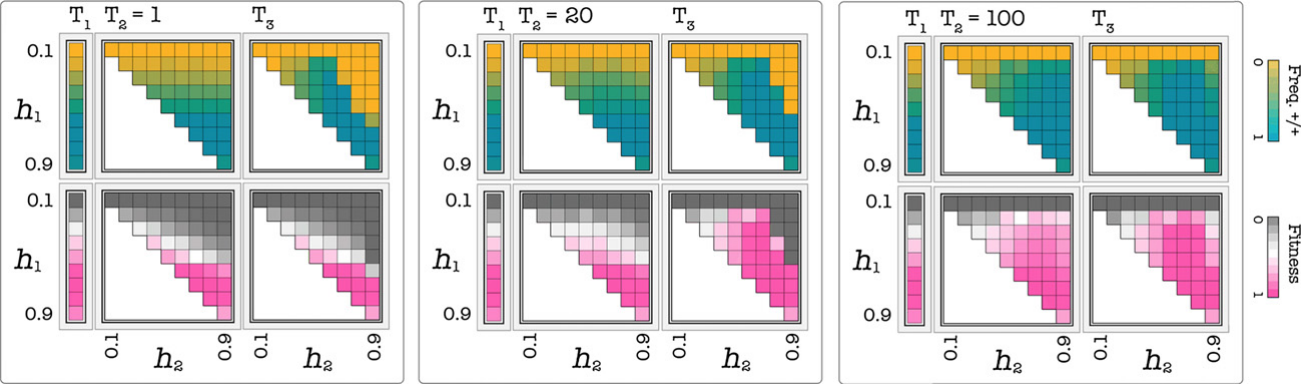
Combined effect of the amount and speed of change. Each cell in each plot shows the frequency of +/+ cells or the average fitness of the population (its growth rate) after T_i_ generations per cell, as a function of h_1_ (the threshold before the treatment) and h_2_ (the threshold after the treatment), for s = 20 and c = 0.01. T_1_=T_3_ = 1000; before T_1_ h_2_=h_1._ A therapy that increases the threshold is effective only when the initial threshold is low, the new threshold is high enough and the shift to the new threshold is fast enough.

The speed of the transition from the original to the new threshold is also essential for the success of a therapy that targets growth factors. While a fast transition to the new threshold can lead to a successful, stable therapy, a slower delivery can lead to relapse (Fig. 8). In summary, therapies are only effective when the initial threshold is low and the increase in threshold is substantial, and if the transition to the new threshold is fast enough (Fig. 9).

#### Dynamics of the evolution of resistance

**Figure 10:**
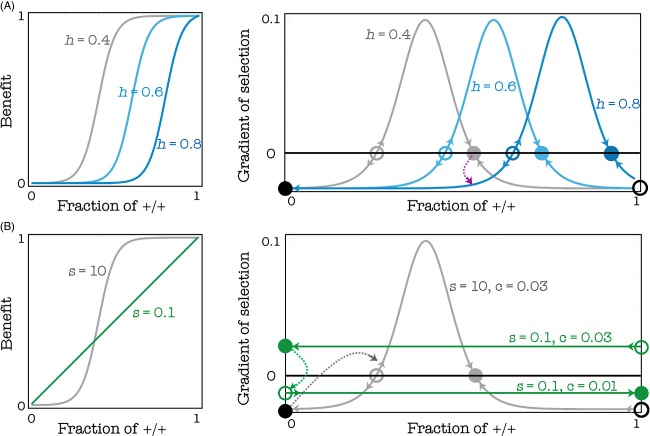
Dynamics of the evolution of resistance to therapies that target growth factors. In a well-mixed population, the gradient of selection determines the direction of the dynamics: where it is positive, the frequency of producers increases; where it is negative it decreases; equilibria (empty circle: unstable; full circle: stable) are found where the gradient of selection is zero (A) Targeting growth factors directly increases the threshold (h) of the public goods game. The therapy is successful (the +/+ cells go extinct) if the new unstable equilibrium is above the original stable equilibrium; if this is not the case, the system will move to the new internal equilibrium. (c = 0.01, s = 10, *n *= 50) (B) If the population is at the pure −/− equilibrium, a mutant +/+ can only invade if the cost declines below the gradient of selection, if the benefit function is linear; if the benefit is nonlinear, random fluctuations can allow small clusters of +/+ to invade and thus allow the population to reach the internal stable equilibrium. (h = 0.5, *n *= 50)

The logic of these two effects (magnitude and speed of the shift in threshold) can be understood by looking at the gradient of selection (Fig. 10 shows the logic for a large, well-mixed population, but the logic is the same in finite populations). The therapy is successful (the +/+ cells go extinct) if and only if the new (posttherapy) unstable equilibrium is above the original (pretherapy) stable equilibrium; if this is not the case, the system will move to the new stable equilibrium. This can happen for two reasons: either the increase in h is not large enough; or the increase is slow enough that the current, transient stable equilibrium remains within the basin of attraction of the new, transient stable equilibrium until the change is completed (Fig. 10A). Note that the evolution of resistance is, therefore, more likely for low values of c (the production cost) and s (the steepness of the benefit function).

#### Instability of successful treatments

**Figure 11:**
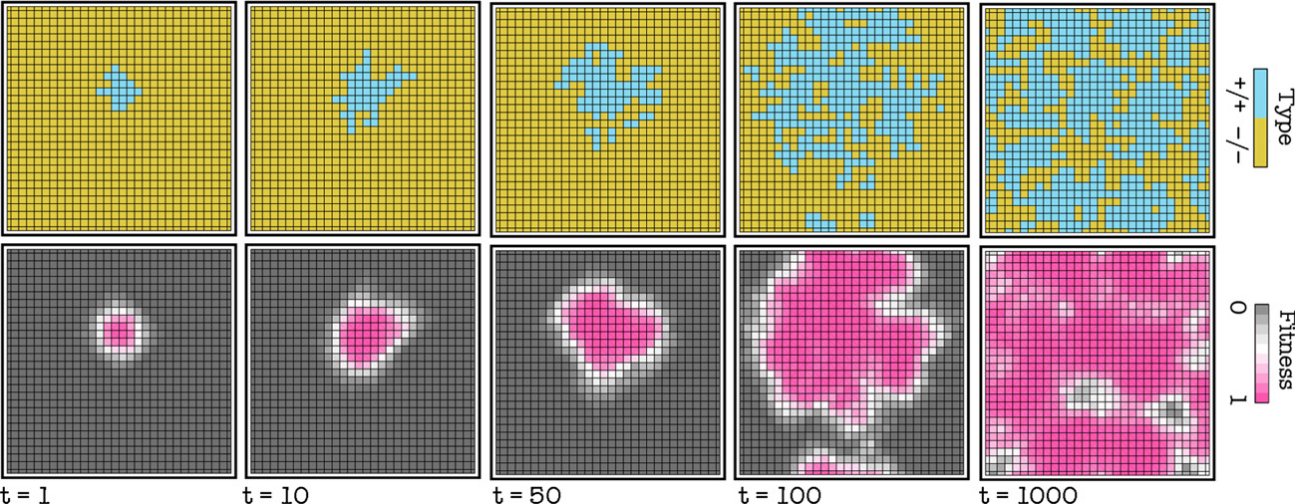
Instability of successful treatments due to random fluctuations. Snapshots (after t generations per cell) of a population initially fixed on −/− in which a mutant +/+ cell line arises and manages to expand. d = 3, c = 0.01, h = 0.5, s = 10, stochastic update.

If the treatment is effective and fast enough that the population does reach the stable pure −/− equilibrium, a mutant +/+ will not be able to invade an infinitely large, well-mixed population. In a finite, structured population, however, it is possible that random fluctuations change the fraction of +/+ cells within a cluster above the unstable equilibrium, which would lead that cluster to the mixed equilibrium in which +/+ and −/− cells coexist (Figs 10B and 11). The opposite effect is also possible, that is, random fluctuations can move the frequency of +/+ at a mixed equilibrium within a group below the unstable internal equilibrium and therefore to the fixation of −/− cells. The relative importance of these two effects depends on the shape of the benefit function, that is on the value of s (the steepness of the benefit function), and on the cost of production (c): low s and c favor the stability of the mixed equilibrium, whereas high s and c make the mixed equilibrium less robust to random fluctuations. An exception to this occurs in the case of very low values of s (that is, for almost linear benefits, similar to the NPD), because such system has only a stable equilibrium: pure +/+ if the cost is low enough, and pure −/− if the cost is low (Fig. 10B); in the latter case, the equilibrium would be immune to invasion by +/+ mutants and therefore arguably stable against the evolution of resistance: a +/+ mutant would only invade if the cost of production decreased.

## Discussion

Analyzing the production of growth factors as a nonlinear public goods game reveals that tumor heterogeneity can be maintained by the frequency-dependent selection that arises as a natural consequence of the fact that growth factors are diffusible, and therefore public goods. Tumor heterogeneity has important implications for diagnosis and treatment. The results help us understand anticancer therapies that attack growth factors, either directly (using drugs like Avastin that target the growth factors) or indirectly (using RNA interference). While it has been suggested that attacking growth factors may be less susceptible to the evolution of resistance (Pepper 2012; Aktipis and Nesse 2013), the results shown here suggest that the issue is not so simple.

The rationale of the analysis is that when one reduces the amount of a growth factor, the immediate result is a sudden reduction in tumor growth, because the threshold necessary to achieve the original benefit is not reached; as a consequence, the growth rate of the tumor immediately declines. At the same time, however, the amount of growth factors necessary for the population to grow increases (because part of them are disrupted by the drug), which changes the dynamics of the system; unfortunately, it changes into the wrong direction: by increasing the threshold, one increase the frequency of producers at equilibrium, which explains relapse simply as the new equilibrium reached by the system under the new conditions. While it is too early to evaluate the efficacy of RNAi treatments, it seems reasonable that even silencing the gene for a growth factor should incur a similar problem and be susceptible to the evolution of resistance.

As pointed out by André and Godelle (2005)and Pepper (2012), therapies that target diffusible factors are a more evolutionarily robust approach than conventional drugs that target cells directly. The logic is that (i) drugs that target growth factors can disrupt cooperation between cells and lead to a pure −/− equilibrium, which will make the population collapse and that (ii) such equilibrium is stable against the invasion of +/+ mutants because the cost paid by the mutant is a private cost, while the benefit it provides is a public benefit. As we have seen, however, (i) requires the therapy to be extremely efficient and fast, and (ii) is not necessarily the case, unless the benefit of the public good is a linear function of the amount of diffusible factors.

As we have shown, the details of the benefit function, diffusion range, cost of production, and update rule that drive the dynamics of growth factor production are critical to determine the type of dynamics and equilibria. More precise theoretical prediction therefore is necessary to understand under what conditions resistance will evolve, including the use of three-dimensional Voronoi graphs to model interactions within the tumor and gradients of diffusion to model the effect of growth factors. Furthermore, the results reported here only apply to growth factors that confer a direct advantage to the tumor, such as factors that protect against apoptosis and promote proliferation; other growth factors, however, act indirectly by inducing the production of other growth factors by stromal cells or by promoting the development of blood vessels. Finally, we have assumed competition due to constant population size, which may describe cancer cell populations that have reached a carrying capacity, but not early stages of tumor growth. Analyzing the dynamics of these cases requires more complex models.

Understanding the production of growth factors as a public goods game suggests that an evolutionarily stable treatment could be achieved through autologous cell therapy (Archetti 2013b): harvesting cancer cells from the patient, knocking out genes coding growth factors in these cells, and reinserting these modified cells inside the tumor. Such therapy, differently from current therapies that target growth factors, would not directly reduce the amount of growth factors produced by the tumor but would change the dynamics of the population. As we have seen, by introducing a critical amount of −/− cells within the tumor, the mixed equilibrium can be destabilised so that the +/+ cells will go extinct; this may lead to the collapse of the tumor due to lack of essential growth factors.
